# Identification of significant periodic genes in microarray gene expression data

**DOI:** 10.1186/1471-2105-6-286

**Published:** 2005-11-30

**Authors:** Jie Chen

**Affiliations:** 1 Department of Mathematics and Statistics, University of Missouri-Kansas City, 5100 Rockhill Road, Kansas City, MO, USA

## Abstract

**Background:**

One frequent application of microarray experiments is in the study of monitoring gene activities in a cell during cell cycle or cell division. A new challenge for analyzing the microarray experiments is to identify genes that are statistically significantly periodically expressed during the cell cycle. Such a challenge occurs due to the large number of genes that are simultaneously measured, a moderate to small number of measurements per gene taken at different time points, and high levels of non-normal random noises inherited in the data.

**Results:**

Based on two statistical hypothesis testing methods for identifying periodic time series, a novel statistical inference approach, the *C&G *procedure, is proposed to effectively screen out statistically significantly periodically expressed genes. The approach is then applied to yeast and bacterial cell cycle gene expression data sets, as well as to human fibroblasts and human cancer cell line data sets, and significantly periodically expressed genes are successfully identified.

**Conclusion:**

The *C&G *procedure proposed is an effective method for identifying statistically significant periodic genes in microarray time series gene expression data.

## Background

Microarray experiments are widely used for gene profiling in different cell lines, various tissues, and conditions (normal versus cancerous). High throughput microarray technologies have made it possible to study problems that range from gene regulation and mRNA stability, to pathways for genetic diseases and the discovery of target subpopulations for drug or other therapies. One frequent application of microarray experiments is in the study of monitoring gene activities in a cell during cell cycle or cell division. A new challenge to statisticians for analyzing the microarray experiments is to identify genes that are statistically significantly periodically expressed during the cell cycle. Such a challenge occurs due to the large number of genes that are simultaneously measured, a moderate to small number of measurements per gene taken at different time points, and high levels of non-normal random noises inherited in the data (Wichert [1]). Several authors, including Spellman [2], Cho [3], Shedden and Cooper [4,5], Whitfield [6] have noticed the presence of cyclicity or periodicity of genes in their microarray data sets and used a number of ways to identify periodically expressed genes in some available yeast and human cell cycle data sets obtained by them. There are some debates concerning the methods those authors used in finding the cyclic genes and how statistically significant those cyclic genes are. Whitfield [6] established a catalog of genes periodically expressed in the human cell cycle via a series of large-scale microarray experiments. They introduced a statistic (periodicity score) for testing the inference of a periodically expressed gene. The method introduced in Whitfield [6], however, may not be effective in identifying multiple periodically expressed genes, as it did not address the multiple comparison issue and hence inflated false discovery rate (FDR). Recently, Wichert [1] proposed to use a graphical device, average periodogram, as an exploratory method to signal the presence of possible periodic genes. They showed through extensive simulations that plotting average periodogram against frequencies reveals the presence of periodic genes in the data set if there is any. They also applied Fisher's exact *G*-test statistic, along with the use of FDR, on the periodogram to screen out statistically significantly periodically expressed genes.

In this paper, another test statistic, the Bartlett's exact *C*-test statistic, for the inference of periodic time series is introduced. By combining both the *G*-statistic and the *C*-statistic, a novel statistical inference approach, the *C&G *procedure, is proposed to effectively screen out statistically significantly periodically expressed. The approach is then applied to yeast and bacterial cell cycle gene expression data sets, as well as to human fibroblasts and human cancer cell line data sets, and significantly periodically expressed genes are successfully identified.

## Results

For testing the null hypothesis of a signal being a normal white noise against the alternative hypothesis of a signal being periodic (see Methods section), a statistical method is to use the periodigrams of the signal (see Methods section for details) to form a test statistic and calculate the p-value of the test statistic. A small p-value, smaller than a predetermined significance level, indicates the significance of the signal being periodic rather than white noise. Fisher [7] proposed a test statistic and derived the null distribution of the Fisher's *G*-statistic. In the context of microarray gene expression data, the observed significance value or p-value for the hypothesis testing of the periodicity of a fixed gene *g*, using *G*-statistic as the test statistic, denoted by pgG
 MathType@MTEF@5@5@+=feaafiart1ev1aaatCvAUfKttLearuWrP9MDH5MBPbIqV92AaeXatLxBI9gBaebbnrfifHhDYfgasaacH8akY=wiFfYdH8Gipec8Eeeu0xXdbba9frFj0=OqFfea0dXdd9vqai=hGuQ8kuc9pgc9s8qqaq=dirpe0xb9q8qiLsFr0=vr0=vr0dc8meaabaqaciGacaGaaeqabaqabeGadaaakeaacqWGWbaCdaqhaaWcbaGaem4zaCgabaGaem4raCeaaaaa@30B2@, can be obtained by



where *ξ*_*g *_is the sample realization of the *G*-statistic value calculated from the Fisher's *G*-statistic (see equation (7) in Methods section) divided by *m*, and *L*(*ξ*_*g*_) is the largest integer less than 1/*ξ*_*g*_.

A more general setting of the hypothesis is to test whether a signal is normal white noise or not. Bartlett [8] proposed a test statistic, the *C*-statistic (see methods), to test for the hypotheses. According to Durbin [9], the p-value for the hypothesis testing of the periodicity of a fixed gene *g *using Bartlett's *C*-statistic as the test statistic, denoted by pgC
 MathType@MTEF@5@5@+=feaafiart1ev1aaatCvAUfKttLearuWrP9MDH5MBPbIqV92AaeXatLxBI9gBaebbnrfifHhDYfgasaacH8akY=wiFfYdH8Gipec8Eeeu0xXdbba9frFj0=OqFfea0dXdd9vqai=hGuQ8kuc9pgc9s8qqaq=dirpe0xb9q8qiLsFr0=vr0=vr0dc8meaabaqaciGacaGaaeqabaqabeGadaaakeaacqWGWbaCdaqhaaWcbaGaem4zaCgabaGaem4qameaaaaa@30AA@, can be found by



where *a*_*g *_= *mC*_*g*_, *C*_*g *_is given in equation (10) of the Methods section, [*a*_*g*_] = *INT*{*a*_*g*_}, and *n *= *m *- 1. Suppose that a large number G
 MathType@MTEF@5@5@+=feaafiart1ev1aaatCvAUfKttLearuWrP9MDH5MBPbIqV92AaeXatLxBI9gBamXvP5wqSXMqHnxAJn0BKvguHDwzZbqegm0B1jxALjhiov2DaebbnrfifHhDYfgasaacH8akY=wiFfYdH8Gipec8Eeeu0xXdbba9frFj0=OqFfea0dXdd9vqai=hGuQ8kuc9pgc9s8qqaq=dirpe0xb9q8qiLsFr0=vr0=vr0dc8meaabaqaciaacaGaaeqabaWaaeGaeaaakeaaimaacaWFhbaaaa@3962@ of genes are simultaneously observed through a microarray experiment, and each gene is measured at a relatively short period, or at sparse intervals of time (say at *N *time points). The researcher is interested in whether any genes are expressed in a periodic pattern of some kind. As high levels of non-normal random noise may present in the data, some visual evidence of periodic gene may be simply due to random noise; and as there are usually a large number of genes (G
 MathType@MTEF@5@5@+=feaafiart1ev1aaatCvAUfKttLearuWrP9MDH5MBPbIqV92AaeXatLxBI9gBamXvP5wqSXMqHnxAJn0BKvguHDwzZbqegm0B1jxALjhiov2DaebbnrfifHhDYfgasaacH8akY=wiFfYdH8Gipec8Eeeu0xXdbba9frFj0=OqFfea0dXdd9vqai=hGuQ8kuc9pgc9s8qqaq=dirpe0xb9q8qiLsFr0=vr0=vr0dc8meaabaqaciaacaGaaeqabaWaaeGaeaaakeaaimaacaWFhbaaaa@3962@ is often from several thousands to several hundreds of thousands), there is a serious concern about the false discovery rate (FDR). Therefore, a multiple comparison approach must be employed to control the FDR level. Recently, Benjamini and Hochberg [10] introduced a practical and powerful approach to multiple testing by controlling the (FDR). This approach is especially useful for multiple hypothesis testing in microarray experiments. It is a step-down type of multiple testing procedure in combination with Bonferroni approach. In light of the p-value, pgC
 MathType@MTEF@5@5@+=feaafiart1ev1aaatCvAUfKttLearuWrP9MDH5MBPbIqV92AaeXatLxBI9gBaebbnrfifHhDYfgasaacH8akY=wiFfYdH8Gipec8Eeeu0xXdbba9frFj0=OqFfea0dXdd9vqai=hGuQ8kuc9pgc9s8qqaq=dirpe0xb9q8qiLsFr0=vr0=vr0dc8meaabaqaciGacaGaaeqabaqabeGadaaakeaacqWGWbaCdaqhaaWcbaGaem4zaCgabaGaem4qameaaaaa@30AA@, obtained using the *C*-statistic, the p-value pgG
 MathType@MTEF@5@5@+=feaafiart1ev1aaatCvAUfKttLearuWrP9MDH5MBPbIqV92AaeXatLxBI9gBaebbnrfifHhDYfgasaacH8akY=wiFfYdH8Gipec8Eeeu0xXdbba9frFj0=OqFfea0dXdd9vqai=hGuQ8kuc9pgc9s8qqaq=dirpe0xb9q8qiLsFr0=vr0=vr0dc8meaabaqaciGacaGaaeqabaqabeGadaaakeaacqWGWbaCdaqhaaWcbaGaem4zaCgabaGaem4raCeaaaaa@30B2@, calculated using the *G*-statistic, and the multiple testing procedure controlling FDR, the following method (called "*C&G *Procedure") is proposed for the selection of periodic gene expressions of the same period:

Step 1: Calculate pgG
 MathType@MTEF@5@5@+=feaafiart1ev1aaatCvAUfKttLearuWrP9MDH5MBPbIqV92AaeXatLxBI9gBaebbnrfifHhDYfgasaacH8akY=wiFfYdH8Gipec8Eeeu0xXdbba9frFj0=OqFfea0dXdd9vqai=hGuQ8kuc9pgc9s8qqaq=dirpe0xb9q8qiLsFr0=vr0=vr0dc8meaabaqaciGacaGaaeqabaqabeGadaaakeaacqWGWbaCdaqhaaWcbaGaem4zaCgabaGaem4raCeaaaaa@30B2@, and pgC
 MathType@MTEF@5@5@+=feaafiart1ev1aaatCvAUfKttLearuWrP9MDH5MBPbIqV92AaeXatLxBI9gBaebbnrfifHhDYfgasaacH8akY=wiFfYdH8Gipec8Eeeu0xXdbba9frFj0=OqFfea0dXdd9vqai=hGuQ8kuc9pgc9s8qqaq=dirpe0xb9q8qiLsFr0=vr0=vr0dc8meaabaqaciGacaGaaeqabaqabeGadaaakeaacqWGWbaCdaqhaaWcbaGaem4zaCgabaGaem4qameaaaaa@30AA@ according to equations (1) and (2), respectively, for *g *= 1, ..., G
 MathType@MTEF@5@5@+=feaafiart1ev1aaatCvAUfKttLearuWrP9MDH5MBPbIqV92AaeXatLxBI9gBamXvP5wqSXMqHnxAJn0BKvguHDwzZbqegm0B1jxALjhiov2DaebbnrfifHhDYfgasaacH8akY=wiFfYdH8Gipec8Eeeu0xXdbba9frFj0=OqFfea0dXdd9vqai=hGuQ8kuc9pgc9s8qqaq=dirpe0xb9q8qiLsFr0=vr0=vr0dc8meaabaqaciaacaGaaeqabaWaaeGaeaaakeaaimaacaWFhbaaaa@3962@.

Step 2: Let the ordered pgC
 MathType@MTEF@5@5@+=feaafiart1ev1aaatCvAUfKttLearuWrP9MDH5MBPbIqV92AaeXatLxBI9gBaebbnrfifHhDYfgasaacH8akY=wiFfYdH8Gipec8Eeeu0xXdbba9frFj0=OqFfea0dXdd9vqai=hGuQ8kuc9pgc9s8qqaq=dirpe0xb9q8qiLsFr0=vr0=vr0dc8meaabaqaciGacaGaaeqabaqabeGadaaakeaacqWGWbaCdaqhaaWcbaGaem4zaCgabaGaem4qameaaaaa@30AA@ values be p(1)C,p(2)C,…,p(G)C
 MathType@MTEF@5@5@+=feaafiart1ev1aaatCvAUfKttLearuWrP9MDH5MBPbIqV92AaeXatLxBI9gBamXvP5wqSXMqHnxAJn0BKvguHDwzZbqegm0B1jxALjhiov2DaebbnrfifHhDYfgasaacH8akY=wiFfYdH8Gipec8Eeeu0xXdbba9frFj0=OqFfea0dXdd9vqai=hGuQ8kuc9pgc9s8qqaq=dirpe0xb9q8qiLsFr0=vr0=vr0dc8meaabaqaciaacaGaaeqabaWaaeGaeaaakeaacqWGWbaCdaqhaaWcbaGaeiikaGIaeGymaeJaeiykaKcabaGaem4qameaaOGaeiilaWIaemiCaa3aa0baaSqaaiabcIcaOiabikdaYiabcMcaPaqaaiabdoeadbaakiabcYcaSiablAciljabcYcaSiabdchaWnaaDaaaleaacqGGOaakimaacaWFhbGaeiykaKcabaGaem4qameaaaaa@4C1F@ with corresponding genes g(1)C,g(2)C,…g(G)C
 MathType@MTEF@5@5@+=feaafiart1ev1aaatCvAUfKttLearuWrP9MDH5MBPbIqV92AaeXatLxBI9gBamXvP5wqSXMqHnxAJn0BKvguHDwzZbqegm0B1jxALjhiov2DaebbnrfifHhDYfgasaacH8akY=wiFfYdH8Gipec8Eeeu0xXdbba9frFj0=OqFfea0dXdd9vqai=hGuQ8kuc9pgc9s8qqaq=dirpe0xb9q8qiLsFr0=vr0=vr0dc8meaabaqaciaacaGaaeqabaWaaeGaeaaakeaacqWGNbWzdaqhaaWcbaGaeiikaGIaeGymaeJaeiykaKcabaGaem4qameaaOGaeiilaWIaem4zaC2aa0baaSqaaiabcIcaOiabikdaYiabcMcaPaqaaiabdoeadbaakiabcYcaSiablAciljabdEgaNnaaDaaaleaacqGGOaakimaacaWFhbGaeiykaKcabaGaem4qameaaaaa@4B09@; and let the ordered pgG
 MathType@MTEF@5@5@+=feaafiart1ev1aaatCvAUfKttLearuWrP9MDH5MBPbIqV92AaeXatLxBI9gBaebbnrfifHhDYfgasaacH8akY=wiFfYdH8Gipec8Eeeu0xXdbba9frFj0=OqFfea0dXdd9vqai=hGuQ8kuc9pgc9s8qqaq=dirpe0xb9q8qiLsFr0=vr0=vr0dc8meaabaqaciGacaGaaeqabaqabeGadaaakeaacqWGWbaCdaqhaaWcbaGaem4zaCgabaGaem4raCeaaaaa@30B2@ be p(1)G,p(2)G,…,p(G)G
 MathType@MTEF@5@5@+=feaafiart1ev1aaatCvAUfKttLearuWrP9MDH5MBPbIqV92AaeXatLxBI9gBamXvP5wqSXMqHnxAJn0BKvguHDwzZbqegm0B1jxALjhiov2DaebbnrfifHhDYfgasaacH8akY=wiFfYdH8Gipec8Eeeu0xXdbba9frFj0=OqFfea0dXdd9vqai=hGuQ8kuc9pgc9s8qqaq=dirpe0xb9q8qiLsFr0=vr0=vr0dc8meaabaqaciaacaGaaeqabaWaaeGaeaaakeaacqWGWbaCdaqhaaWcbaGaeiikaGIaeGymaeJaeiykaKcabaGaem4raCeaaOGaeiilaWIaemiCaa3aa0baaSqaaiabcIcaOiabikdaYiabcMcaPaqaaiabdEeahbaakiabcYcaSiablAciljabcYcaSiabdchaWnaaDaaaleaacqGGOaakimaacaWFhbGaeiykaKcabaGaem4raCeaaaaa@4C37@ with corresponding genes g(1)G,g(2)G,…g(G)G
 MathType@MTEF@5@5@+=feaafiart1ev1aaatCvAUfKttLearuWrP9MDH5MBPbIqV92AaeXatLxBI9gBamXvP5wqSXMqHnxAJn0BKvguHDwzZbqegm0B1jxALjhiov2DaebbnrfifHhDYfgasaacH8akY=wiFfYdH8Gipec8Eeeu0xXdbba9frFj0=OqFfea0dXdd9vqai=hGuQ8kuc9pgc9s8qqaq=dirpe0xb9q8qiLsFr0=vr0=vr0dc8meaabaqaciaacaGaaeqabaWaaeGaeaaakeaacqWGNbWzdaqhaaWcbaGaeiikaGIaeGymaeJaeiykaKcabaGaem4raCeaaOGaeiilaWIaem4zaC2aa0baaSqaaiabcIcaOiabikdaYiabcMcaPaqaaiabdEeahbaakiabcYcaSiablAciljabdEgaNnaaDaaaleaacqGGOaakimaacaWFhbGaeiykaKcabaGaem4raCeaaaaa@4B21@.

Step 3: For a given FDR level of *q*, let *i*_*q *_be the largest *i *for which p(i)C≤1Gq
 MathType@MTEF@5@5@+=feaafiart1ev1aaatCvAUfKttLearuWrP9MDH5MBPbIqV92AaeXatLxBI9gBamXvP5wqSXMqHnxAJn0BKvguHDwzZbqegm0B1jxALjhiov2DaebbnrfifHhDYfgasaacH8akY=wiFfYdH8Gipec8Eeeu0xXdbba9frFj0=OqFfea0dXdd9vqai=hGuQ8kuc9pgc9s8qqaq=dirpe0xb9q8qiLsFr0=vr0=vr0dc8meaabaqaciaacaGaaeqabaWaaeGaeaaakeaacqWGWbaCdaqhaaWcbaGaeiikaGIaemyAaKMaeiykaKcabaGaem4qameaaOGaeyizIm6aaSaaaeaacqaIXaqmaeaaimaacaWFhbaaaiabdghaXbaa@433E@, and let *j*_*q *_be the largest *j *for which p(j)G≤jGq
 MathType@MTEF@5@5@+=feaafiart1ev1aaatCvAUfKttLearuWrP9MDH5MBPbIqV92AaeXatLxBI9gBamXvP5wqSXMqHnxAJn0BKvguHDwzZbqegm0B1jxALjhiov2DaebbnrfifHhDYfgasaacH8akY=wiFfYdH8Gipec8Eeeu0xXdbba9frFj0=OqFfea0dXdd9vqai=hGuQ8kuc9pgc9s8qqaq=dirpe0xb9q8qiLsFr0=vr0=vr0dc8meaabaqaciaacaGaaeqabaWaaeGaeaaakeaacqWGWbaCdaqhaaWcbaGaeiikaGIaemOAaOMaeiykaKcabaGaem4raCeaaOGaeyizIm6aaSaaaeaacqWGQbGAaeaaimaacaWFhbaaaiabdghaXbaa@43B5@.

Step 4: The intersection set K={g(1)C,g(2)C,…g(iq)C}∩{g(1)G,g(2)G,…g(jq)G}
 MathType@MTEF@5@5@+=feaafiart1ev1aaatCvAUfKttLearuWrP9MDH5MBPbIqV92AaeXatLxBI9gBaebbnrfifHhDYfgasaacH8akY=wiFfYdH8Gipec8Eeeu0xXdbba9frFj0=OqFfea0dXdd9vqai=hGuQ8kuc9pgc9s8qqaq=dirpe0xb9q8qiLsFr0=vr0=vr0dc8meaabaqaciGacaGaaeqabaqabeGadaaakeaacqWGlbWscqGH9aqpcqGG7bWEcqWGNbWzdaqhaaWcbaGaeiikaGIaeGymaeJaeiykaKcabaGaem4qameaaOGaeiilaWIaem4zaC2aa0baaSqaaiabcIcaOiabikdaYiabcMcaPaqaaiabdoeadbaakiabcYcaSiablAciljabdEgaNnaaDaaaleaacqGGOaakcqWGPbqAdaWgaaadbaGaemyCaehabeaaliabcMcaPaqaaiabdoeadbaakiabc2ha9jabgMIihlabcUha7jabdEgaNnaaDaaaleaacqGGOaakcqaIXaqmcqGGPaqkaeaacqWGhbWraaGccqGGSaalcqWGNbWzdaqhaaWcbaGaeiikaGIaeGOmaiJaeiykaKcabaGaem4raCeaaOGaeiilaWIaeSOjGSKaem4zaC2aa0baaSqaaiabcIcaOiabdQgaQnaaBaaameaacqWGXbqCaeqaaSGaeiykaKcabaGaem4raCeaaOGaeiyFa0haaa@5FE9@ then contains all the statistically significantly periodically expressed genes (of the same period). The difference set D={g(1)C,g(2)C,…g(iq)C}\K
 MathType@MTEF@5@5@+=feaafiart1ev1aaatCvAUfKttLearuWrP9MDH5MBPbIqV92AaeXatLxBI9gBaebbnrfifHhDYfgasaacH8akY=wiFfYdH8Gipec8Eeeu0xXdbba9frFj0=OqFfea0dXdd9vqai=hGuQ8kuc9pgc9s8qqaq=dirpe0xb9q8qiLsFr0=vr0=vr0dc8meaabaqaciGacaGaaeqabaqabeGadaaakeaacqWGebarcqGH9aqpcqGG7bWEcqWGNbWzdaqhaaWcbaGaeiikaGIaeGymaeJaeiykaKcabaGaem4qameaaOGaeiilaWIaem4zaC2aa0baaSqaaiabcIcaOiabikdaYiabcMcaPaqaaiabdoeadbaakiabcYcaSiablAciljabdEgaNnaaDaaaleaacqGGOaakcqWGPbqAdaWgaaadbaGaemyCaehabeaaliabcMcaPaqaaiabdoeadbaakiabc2ha9jabcYfaCjabdUealbaa@48D3@ then contains possible periodic genes with different periods, or of other patterns other than periodic.

A natural question that might come up is: What is the FDR level of the identified periodic genes contained in set K? A straightforward proof leads to the conclusion that the FDR level of the identified periodic genes contained in set *K *of step 4 in the C&G Procedure is at most *q*. In other words, by using this procedure, the FDR level is not inflated. The application of the *C&G *Procedure is illustrated in the following four examples.

### Analysis of the bacterial cell cycle data

**Figure 1:**
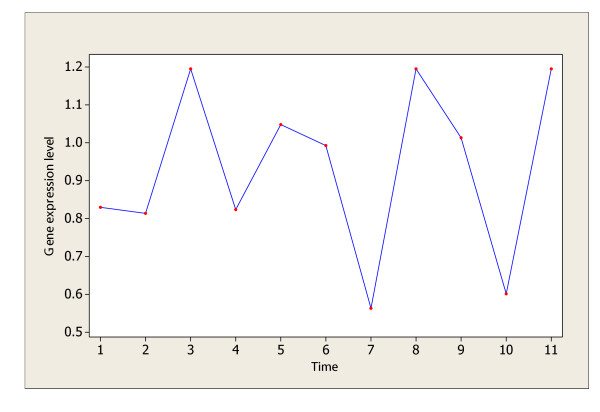
The gene (ORF00082) in Laub data that is not considered periodic in this paper.

The gene expression data from synchronized bacterium *Caulobacter crescentus *cells (Laub [11]) is analyzed for possible periodically expressed genes using the procedure proposed in this paper. The data can be downloaded from the Bacterial cell cycle data website [12]. It contains information on 1474 genes over 11 equally spaced time points (with a time interval of 15 minutes). There are 533 genes identified as cell-cycle regulated genes in Laub [11], while for the same data Wichert [1] claims that only 44 genes are cyclic genes at FDR level of 0.05. Using the C&G Procedure of this paper, it is found out that the *C*-statistic identifies 166 genes as significant non-white noise expressions (including possible cell-cycle regulated genes) and the G-statistic identifies 44 such genes; their intersection set contains 43 significant cell-cycle regulated genes. Therefore, we claim that there are 43 significant periodic genes (of the same period) at FDR level of 0.05. This conclusion matches very well with that of Wichert *et al*. (2004). The one gene which is considered as a periodic gene in Wichert [1] but not as such a gene in this paper is ORF00082 (ABC transporter, ATP-binding protein), whose expression plot against the time is given in Figure 1. Clearly, Figure 1 shows a fluctuation pattern rather than a periodic pattern. The ATP-binding protein gives general function prediction only and its biological function is poorly categorized according to the archive information provided on the National Center for Biotechnology Information (NCBI) website [13].

### Analysis of the yeast cell cycle data

In the second example, the gene expression data sets from the well-known yeast *Saccharomyces cerevisiae *microarray experiments of Spellman [2] are analyzed for the identification of significantly periodically expressed genes. The data sets can be downloaded from the Yeast cell cycle data website [14]. These four data sets were produced by three different cell cycle synchronization techniques: alpha factor arrest (producing the "alpha" gene expression data), temperature arrest (producing "cdc15" and "cdc28" gene expression data sets), and elutriation synchronization (producing the "elution" data set). The alpha data set contains complete information on 4489 genes over 18 equally spaced time points (with a time interval of 7 minutes). Using the C&G Procedure, it is found out that the C-statistic identifies 1188 genes as significant non-white noise expressions (including possible cell-cycle regulated genes) and the G-statistic identifies 473 such genes, their intersection set contains 471 significant cell-cycle regulated genes. Therefore, we claim that there are at least 471 significant periodic genes (of the same period) at FDR level of 0.05 in the alpha experiment data, and there are additional 717 genes in set D that are possibly periodic with different periods, or of other patterns other than periodic.

**Figure 2:**
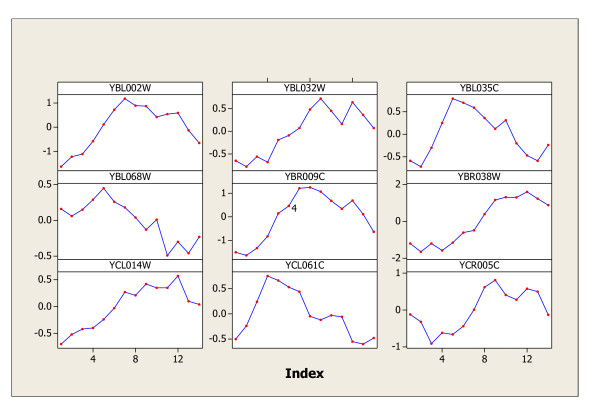
The nine most significant periodic genes in Elution data.

**Figure 3:**
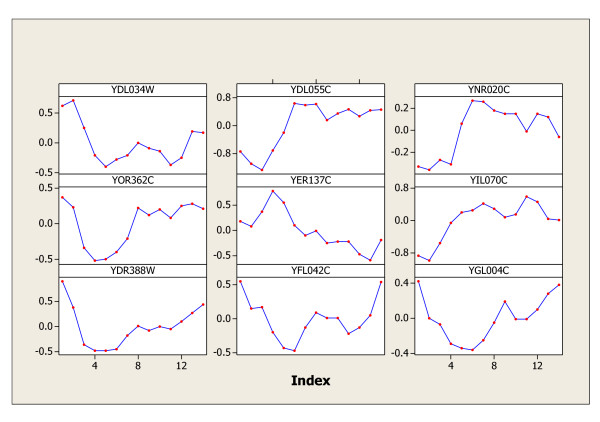
The nine most significant genes in set D for Elution data.

The same procedure is applied to the cdc15, cdc28, and elution data sets, and the genes identified by both statistics, their intersection set K, and the difference set D are summarized in Table 1. Spellman [2] originally identified 800 cell-cycle genes in all of the four experiments (alpha, cdc15, cdc28, and elution), while Wichert [1] claimed 468 cyclic genes in alpha, 766 cyclic genes in cdc15, 105 in cdc28, and 193 in elution. The periodic genes found by the *C&G *procedure are obviously in agreement with the findings in Spellman [2] to some extent, and agree more with the findings in Wichert [1], but not completely agree with theirs. The genes identified in the difference set *D *worth further investigation by biologists as they may lead to new interesting discoveries. Furthermore, the results found in this paper are certainly improvements over their discoveries of periodic genes. The nine most significant periodic genes in elution data are graphed in Figures 2 for illustration purpose. The nine most significant genes (YDL034W, YDL055C, YNR020C, YOR362C, YER137C, YIL070C, YDR388W, YFL042C, and YGL004C) in set D of elution data are graphed in Figure 3. The patterns of these nine genes in Figure 3 certainly represent a mixture of expressions of periodic, periodic with different period(s), or of other patterns other than periodic. The genetic footprinting of YDL034W, YDL055C, YNR020C, YIL070C, YDR388W, YFL042C, and YGL004C reveal apparent moderate growth defect on YPD after 20 generations according to the archive information provided on the yeast genome website [15]. This means that the expressions of these seven genes show gradual decade patterns rather than random patterns. Hence, our findings in set D really make biological sense. Gene YOR362C participates in endopeptidase activity and the molecular function of gene YER137C is still unknown.

**Table 1:** Number of Significant Periodic Genes Identified by C-statistics, G-statistic, Intersection Set K, and Difference Set D

Cell type	Experiment	*N*	*G*	*N*_*C*_	*N*_*G*_	*N*_*K*_	*N*_*D*_
*C. crescentus*	bacteria	11	1474	166	44	43	123

Yeast	alpha	18	4489	1188	473	471	717
Yeast	cdc15	24	4381	1636	788	779	857
Yeast	cdc28	17	1383	292	27	27	265
Yeast	Elution	14	5766	1056	769	695	361

Human fibroblasts	N2	12	7077	1	2	1	0
Human fibroblasts	N3	12	7077	2	0	0	2

Human HeLa	Score1	12	15536	44	7	6	38
Human HeLa	Score2	26	16287	1351	154	153	1198
Human HeLa	Score3	48	41508	9702	6117	5770	3932
Human HeLa	Score4	19	40815	52	52	17	35
Human HeLa	Score5	9	35871	5	1	0	1

*N*: number of time points; *G*: number of probe sets; *N*_*C*_: number of significant genes picked up by C-statistic; *N*_*G*_: number of significant genes picked up by G-statistic; *N*_*K*_: number of significant periodic genes picked up by the intersection set K; *N*_*D*_: number of significant other periodic genes or other patterned genes picked-up by the difference set D.

### Analysis of human fibroblasts data

**Figure 4:**
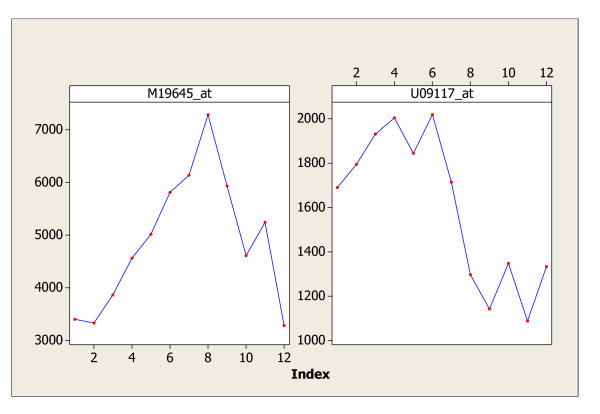
The two genes in set D of N3 data.

In this example, the microarray data on the transcriptional profiling of the cell cycle in human fibroblasts will be analyzed. The experiments and data sets are reported in Cho [3]. The data is available at the Human fibroblasts data website [16]. There are two data sets resulted from experiment N2 and experiment N3 with 12 time points and 7077 probe sets. There were approximately 700 genes that were claimed as periodic genes in Cho [3]. The claim was based on clustering and pattern matching as described by Cho [3]. Shedden and Cooper [4] had doubts about the biological grounds of the data analysis results which were claimed to be statistically significant in Cho [3]. Wichert [1] found no significant periodic genes in these two data sets. Applying the C&G Procedure of this paper to N2 data set, it is found out that the *C*-statistic identifies 1 gene as significant non-white noise expressions (including possible cell-cycle regulated genes) and the *G*-statistic identifies 2 such genes; their intersection set contains 1 significant cell-cycle regulated gene. Therefore, we claim that there is one significant periodic gene at FDR level of 0.05 in the N2 data set. Similarly, for the N3 data set, the *C*-statistic identifies 2 genes as significant non-white noise expressions (including possible cell-cycle regulated genes), and the *G*-statistic identifies 0 such gene; their intersection set contains 0 gene. Therefore, we claim that there is no significant periodic gene in the N3 data set. This conclusion matches very well with that of Wichert [1]. What is more interesting is that the two genes *M*19645_*a*_*t *(or HSPA5) and *U*09117_*a*_*t *(or PLCD1) identified in set D (expressions are shown in Figure 4) certainly show some patterns which require further biological investigations. Gene HSPA5 belongs to the heat shock protein 70 family and probably plays a role in facilitating the assembly of multimeric protein complexes inside the ER, while gene PLCD1 participates in a protein coding process in the organ Bos Taurus (information available at NCBI).

### Analysis of human cancer cell line data

**Figure 5:**
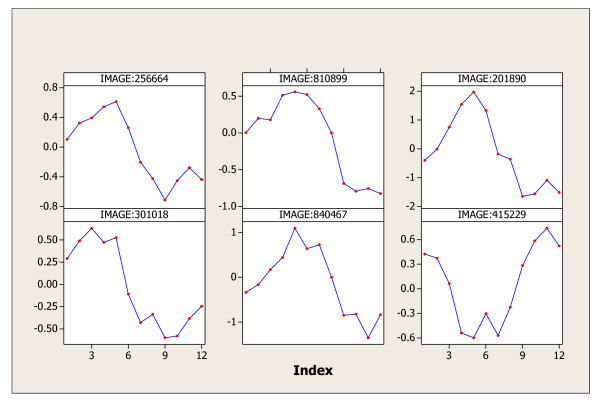
The six significant periodic genes in set K of Score1 data.

In this last example, the human cancer cell line profiling data sets resulted from large-scale microarray experiments given in Whitfield [6] will be analyzed by using the C&G Procedure. The data sets can be downloaded from the Human cancer cell line data website [17]. There were 5 experiments conducted using three different cell cycle synchronization methods: a double thymidine block method (resulting in three data sets Score 1, Score2, and Score3); thymidine followed by arrest in mitosis with nocodazole (resulting in data set Score4) ; and mitotic shake-off using an automated cell shake (resulting in data set Score5). The C&G procedure is applied to these five data sets, and the findings are also given in Table 1. In particular, the six significant periodic genes identified in set K of Score1 data are graphed in Figure 5; and their periodic patterns are quite evident. These six genes have gene symbols: H2AFX, CKS1, BIRC3, STK9, FLJ11259, and VAV3, respectively. According to the NCBI website, H2AFX encodes a member of the histone H2A family, and generates two transcripts through the use of the conserved stem-loop termination motif, and the polyA addition motif. Gene CKS1 is a protein coding gene in a human cell division control protein family. The BIRC3 protein coding gene is the inhibitor of apoptosis protein 1. STK9 is also a protein coding gene but its biological process is still unknown. FLJ11259 is a protein coding gene foe a hypothetical protein. VAV3 regulates the B cell responses by promoting the sustained production of PIP3 and thereby calcium flux. Therefore, close biological research on these six genes should be very worthy according to their detected patterns found by the C&G procedure. The data sets analyzed by C-statistic, G-statistic, and C&G Procedure in all above examples are summarized in Table 1. It is noted that the genes in Set K of Table 1 are claimed as periodic genes (of the same period) by the C&G procedure. The difference set D contains genes of periodic, periodic with different period(s), or of other patterns other than periodic. Genes in set D worth biologists' further study and discovery. Table 2 gives the comparison of the results obtained by C&G Procedure to the results obtained by the researchers who originally conducted those experiments, and to the results obtained by Wichert [1].

**Table 2:** Number of Periodic Genes Identified by the Original Experimenters, Wichert et al. (2004), and Chen

Cell type	Experiment	Experimenter	Wichert *et al*. (2004)	Chen
*C. crescentus*	bacteria	Laub *et al*. (2000), identified 553 periodic genes	44	43

Yeast	alpha	Spellman *et al*. (1998),	468	471
Yeast	cdc15	total of 800 periodic genes	766	779
Yeast	cdc28	identified in all of these four	105	27
Yeast	Elution	yeast cell cycle experiments	193	695

Human fibroblasts	N2	Cho *et al*. (2001), 700 periodic	0	1
Human fibroblasts	N3	genes identified in N2 and N3	0	0

Human HeLa	Score1	Whitfield *et al*. (2002),	0	6
Human HeLa	Score2	total of 800+ periodic genes	134	153
Human HeLa	Score3	identified in these five	6043	5770
Human HeLa	Score4	Human Cancer cell line	56	17
Human HeLa	Score5	experiments	0	0

## Discussion

Regarding both of the test statistics, several points need to be addressed.

First of all, the *G*-statistic is testing for the significance of the maximum periodogram. When the result is significant, the message conveyed to us is that the maximum periodogram is significant with the possible cause of the underlying model being periodic. On the other hand, the *C*-statistic utilizes a sort of standardized cumulative periodograms, and considers all periodograms' contributions towards the periodicity of the underlying model. Therefore, these two statistics are not exactly the same. Secondly, although both *G*-statistic and *C*-statistic can be used as test statistics for searching periodicity in a time series, the *G*-statistic method is more specific and the *C*-statistic method is broader in the sense that the alternative hypothesis to the null hypothesis is rather vague. In other words, for a fixed gene *g*, when the p-value pgG
 MathType@MTEF@5@5@+=feaafiart1ev1aaatCvAUfKttLearuWrP9MDH5MBPbIqV92AaeXatLxBI9gBaebbnrfifHhDYfgasaacH8akY=wiFfYdH8Gipec8Eeeu0xXdbba9frFj0=OqFfea0dXdd9vqai=hGuQ8kuc9pgc9s8qqaq=dirpe0xb9q8qiLsFr0=vr0=vr0dc8meaabaqaciGacaGaaeqabaqabeGadaaakeaacqWGWbaCdaqhaaWcbaGaem4zaCgabaGaem4raCeaaaaa@30B2@ is small compared with a predetermined significance level, the conclusion that this gene is a significant periodic gene according to the *G*-statistic can be reached; however, when the p-value pgC
 MathType@MTEF@5@5@+=feaafiart1ev1aaatCvAUfKttLearuWrP9MDH5MBPbIqV92AaeXatLxBI9gBaebbnrfifHhDYfgasaacH8akY=wiFfYdH8Gipec8Eeeu0xXdbba9frFj0=OqFfea0dXdd9vqai=hGuQ8kuc9pgc9s8qqaq=dirpe0xb9q8qiLsFr0=vr0=vr0dc8meaabaqaciGacaGaaeqabaqabeGadaaakeaacqWGWbaCdaqhaaWcbaGaem4zaCgabaGaem4qameaaaaa@30AA@ is small, only the claim that this gene is not a white noise (might be of periodic, periodic with different period, or of other patterns other than periodic) according to the *C*-statistic can be drawn. Hence, one can anticipate that the *C*-statistic will pick up more significant genes than the *G*-statistic. This is valuable, especially in expensive microarray experiments, because the biologist can use the information to possibly discover genes that are of different periods, or of other pattern which they have not encountered before. Thirdly, from the definitions of the two statistics (see Methods), we can easily establish that

*G_g _*≥ 1,0 ≤ *C_g _*≤ 1, and *G_g _*≥*C_g_*.     (3)

Then, the fact that *G*_*g *_is great than its threshold value does not necessarily imply that *C*_*g *_is greater than its threshold value, and vise versa. In other words, from the fact given by (3), it is clear that these two statistics are not equivalent in general; there are times, however, that both tests overlap with each other. This is not surprising because the *G*-statistic is constructed for testing normal white noise versus periodic function, and the *C*-statistic method is broader in the sense that the alternative hypothesis to the null hypothesis is rather vague. One might think that the set of periodic signals identified by the *G*-statistic is contained in the set of genes identified by the *C*-statistic. It is not necessarily true for the reasons mentioned here in this section.

Furthermore, the *G*-statistic method is sensitive to the departure from normality as pointed in Davis [18] and Wilks [19]. Hence, when the normality assumption on the random errors is violated, the null distribution of the *G*-statistic will not be true in general and the p-value in (1) could be very wrong. The *C*-statistic method is insensitive to the departure of normality as pointed out in Durbin [9]. The two statistics can then be served as constraints for each other in order to effectively search for true periodic genes.

Moreover, the behavior of the *C*-statistic method, the *G*-statistic method, and the C&G Procedure for identifying periodic signals is empirically studied by means of the following simulation studies. To investigate the power of the three methods under different noise conditions, a sine signal mixed with a normal white noise (with the ratio of amplitude of signal to noise being 1 : 1) on 20 time points is simulated 10,000 times, and the frequency that each of the three methods rejects the null hypothesis (at the false positive rate of 0.05), or identifies the signal as periodic, is recorded. Similarly, a sine signal mixed with a skewed noise (a chi-square distribution with 1 degree of freedom) on 20 time points is simulated 10,000 times, and the frequency that each of the three methods rejects the null hypothesis is recorded. The empirical power of each method is hence obtained and listed in Table 3. From Table 3, we conclude that the empirical powers of all three methods increase if the noise is improved from skewed distribution to normal distribution. Under each noise condition, the *C*-statistic method has higher power than that of the other two methods. The power of *C&G *Procedure is about the same as the *G*-statistic method. When the periodic signal is stronger than the normal white noise (with the ratio of amplitude of signal to noise being 9 : 8, 10 : 8, 11 : 8, 12 : 8, respectively), our simulation (10,000 times) of such signals on 20 time points shows that all three methods have high powers (see Table 4). This is a very good property of all three methods. Next, to study the effectiveness of the methods in identifying true periodic signals when the data is noisy, weaker sine signals mixed with a stronger normal white noise (with the ratio of amplitude of signal to noise being 7 : 8, 6 : 8, 5 : 8, 4 : 8 or 1 : 2, respectively) on 20 time points are simulated for 10,000 times each. The empirical powers of the *C*-statistic, the *G*-statistic, and the *C*&*G *Procedure are given in Table 5. We conclude from Table 5 that the empirical power of the *C*-statistic method is always higher than the other methods, and the empirical power of *C*&*G *Procedure is about the same or compatible with the *G*-statistic method when the data is even very noisy (signal to noise amplitude ratio being 1:2). The power of all methods decreases when the noise dominants the true periodic signal more and more (see the powers from row 2 to row 5 of Table 5). As there usually are strong and weak signals in a large gene expression dataset, knowing the behavior of all three methods under these situations helps the biologist to choose a right searching tool for analyzing their experimental data. Although these simulation studies show that the power of the *C*-statistic is higher than that of the other two methods, we need to investigate the empirical type I error rate, or false positive rate, of these three methods. For this purpose, a sequence of 20 normal observations (without any periodic signals) is simulated 10,000 times, the frequency that each of the three methods considers the observations as periodic signals (at the priori false positive rate of 0.05) is recorded. Similarly, a sequence of 20 Chi-square (1 degree of freedom) observations (without any periodic signals) and a sequence of 20 observations (without any periodic signals) from uniform (0,1) distribution are simulated 10,000 times, and the similar frequencies are recorded. Then the empirical false positive rates of the three methods are obtained and summarized in Table 6. It is clear that the false positive rate of the *C*-statistic is the highest and that of the *C*&*G *Procedure is the least under each noise scenario. All simulation studies together indicate that to maintain a stable and relatively high power and to minimize the false positive rate, the *C*&*G *Procedure is a right choice. Thus, the advantage of using the proposed *C*&*G *Procedure emerges. The simulation, as well as all calculations in previous sections, is carried out using Matlab and Mintab 14.

**Table 3:** Empirical power of *C*, *G*, and *C&G *with the ratio of amplitude of signal to noise being 1 : 1

Signal type	*C*	*G*	*C&G*
sine signal with skewed noise	81.66%	75.25%	75.23%
sine signal with normal white noise	99.09%	97.57%	97.57%

**Table 4:** Empirical power of *C*, *G*, and *C&G *on stronger signals

The ratio of amplitude of signal to noise	*C*	*G*	*C&G*
9:8	99.78%	99.50%	99.50%
10:8	99.97%	99.93%	99.93%
11:8	99.99%	99.99%	99.99%
12:8	100%	100%	100%

**Table 5:** Empirical power of *C*, *G*, and *C&G *on weaker signals

The ratio of amplitude of signal to noise	*C*	*G*	*C&G*
7:8	96.00%	91.72%	91.72%
6:8	87.39%	78.40%	78.40%
5:8	71.95%	56.87%	56.82%
4:8	51.03%	34.30%	34.01%

**Table 6:** Empirical false positive rate of *C*, *G*, and *C&G*

noise type	*C*	*G*	*C&G*
normal	12.3%	6.45%	4.29%
uniform	13.23%	7.60%	4.70%
Chi-square	7.32%	2.32%	1.36%

Finally, as the null distributions of these two statistics are all exact distributions, they work well (as long as the underlying assumptions are met) for any sample size (small or large). This characteristic makes both tests very valuable to microarray data sets as the observations obtained for each gene is usually not large in a microarray experiment.

## Conclusion

In this paper a statistical C&*G *Procedure is proposed for identifying significantly periodically expressed genes for a desired FDR level *q*. This approach uses both Bartlett's *C*-statistic and Fisher's *G*-statistic to secure the actual periodic genes existing in a microarray data set. As the searching process is also a multiple testing procedure, the FDR level is used to assure that the overall false discover rate for the whole procedure is at most *α*. The *G*-statistic does assume that the sequence is Gaussian, this may not be the case for any microarray data set. Nevertheless, a log-transformed expression data usually can satisfy the Gaussian assumption. The *C*-statistic is more robust towards the violation of Gaussian assumption. The advantage of the *C*&*G *Procedure thus emerges. Although the gene expression sequences in a microarray data set are usually correlated, the approach of the multiple testing with controlled FDR level does not rely on independence assumption heavily according to Benjamini and Hochberg [10]. Therefore, this *C*&*G *Procedure is a promising statistical tool for finding significantly periodically expressed genes (of the same period) in a microarray data set. Other issues, such as the analysis of data measured in unevenly spaced time intervals and the size of each sequence needed for valid statistical analysis, will be topics of future investigations in order to more effectively search for significantly periodically expressed genes in a microarray data set.

## Methods

Suppose that a time series is observed and one concern is the possible periodicity of this time series. To be specific in the context of gene expressions observed at time *t *for any fixed gene *g*, we denote the time series (or gene expression observed in a time course) by *Y*_*g*_(*t*) for *t *= 1, ..., *N *and *g *= 1, ..., G
 MathType@MTEF@5@5@+=feaafiart1ev1aaatCvAUfKttLearuWrP9MDH5MBPbIqV92AaeXatLxBI9gBamXvP5wqSXMqHnxAJn0BKvguHDwzZbqegm0B1jxALjhiov2DaebbnrfifHhDYfgasaacH8akY=wiFfYdH8Gipec8Eeeu0xXdbba9frFj0=OqFfea0dXdd9vqai=hGuQ8kuc9pgc9s8qqaq=dirpe0xb9q8qiLsFr0=vr0=vr0dc8meaabaqaciaacaGaaeqabaWaaeGaeaaakeaaimaacaWFhbaaaa@3962@. To model *Y*_*g*_(*t*) with periodicity, we can assume:

*Y_g_*(*t*) = *f_g_*(*t*) + *ε_gt_*,

where *f*_*g*_(*t*) is a periodic function with a smallest positive period *T*_*g *_for gene *g*, that is *f*_*g*_(*t *+ *T*_*g*_) = *f*_*g*_(*t*) for all *t*; and *ε*_*gt *_is a sequence of non-observable random errors with mean 0 and homogenous variance *σ*^2 ^for all *g *and *t*. For a fixed gene *g*, we can specifically assume that a time series gene expression is well represented by

*Y_g_*(*t*) = *μ *+ *A *cos (*ωt*) + *B *sin(*ωt*) + *ε_gt_*,

where *A*, *B*, and *μ *(known) are constants, *ω *is of the form 2*πk*/*N*, for *k *= 0,1, ..., *m*, with *m *= (*N *- 1)/2 for *N *odd and *m *= *N*/2 for *N *even. Given a finite realization of the time series gene expressions *y*_*g*_(*t*) (sample values or microarray expressions obtained from the experiment), we can then view *y*_*g*_(*t*) as represented by

yg(t)=2y¯g+∑k=1m[ag,kcos⁡(ωkt)+bg,ksin⁡(ωkt)],
 MathType@MTEF@5@5@+=feaafiart1ev1aaatCvAUfKttLearuWrP9MDH5MBPbIqV92AaeXatLxBI9gBaebbnrfifHhDYfgasaacH8akY=wiFfYdH8Gipec8Eeeu0xXdbba9frFj0=OqFfea0dXdd9vqai=hGuQ8kuc9pgc9s8qqaq=dirpe0xb9q8qiLsFr0=vr0=vr0dc8meaabaqaciGacaGaaeqabaqabeGadaaakeaacqWG5bqEdaWgaaWcbaGaem4zaCgabeaakiabcIcaOiabdsha0jabcMcaPiabg2da9iabikdaYiqbdMha5zaaraWaaSbaaSqaaiabdEgaNbqabaGccqGHRaWkdaaeWbqaaiabcUfaBjabdggaHnaaBaaaleaacqWGNbWzcqGGSaalcqWGRbWAaeqaaOGagi4yamMaei4Ba8Maei4CamNaeiikaGIaeqyYdC3aaSbaaSqaaiabdUgaRbqabaGccqWG0baDcqGGPaqkcqGHRaWkcqWGIbGydaWgaaWcbaGaem4zaCMaeiilaWIaem4AaSgabeaakiGbcohaZjabcMgaPjabc6gaUjabcIcaOiabeM8a3naaBaaaleaacqWGRbWAaeqaaOGaemiDaqNaeiykaKIaeiyxa0LaeiilaWcaleaacqWGRbWAcqGH9aqpcqaIXaqmaeaacqWGTbqBa0GaeyyeIuoaaaa@639B@

where *ω_k _*= 2*πk*/*N*, for *k *= 0,1, ..., *m*, , and



for *k *= 1, ..., *m *and g = 1, ..., G
 MathType@MTEF@5@5@+=feaafiart1ev1aaatCvAUfKttLearuWrP9MDH5MBPbIqV92AaeXatLxBI9gBamXvP5wqSXMqHnxAJn0BKvguHDwzZbqegm0B1jxALjhiov2DaebbnrfifHhDYfgasaacH8akY=wiFfYdH8Gipec8Eeeu0xXdbba9frFj0=OqFfea0dXdd9vqai=hGuQ8kuc9pgc9s8qqaq=dirpe0xb9q8qiLsFr0=vr0=vr0dc8meaabaqaciaacaGaaeqabaWaaeGaeaaakeaaimaacaWFhbaaaa@3962@. For the testing of periodicity related hypotheses of a time series, the periodogram of gene *g *is denned as

Ig,n(ωk)=2N|∑t=1NYg(t)e−iωkt|2=N2(ag,k2+bg,k2),     (4)
 MathType@MTEF@5@5@+=feaafiart1ev1aaatCvAUfKttLearuWrP9MDH5MBPbIqV92AaeXatLxBI9gBaebbnrfifHhDYfgasaacH8akY=wiFfYdH8Gipec8Eeeu0xXdbba9frFj0=OqFfea0dXdd9vqai=hGuQ8kuc9pgc9s8qqaq=dirpe0xb9q8qiLsFr0=vr0=vr0dc8meaabaqaciGacaGaaeqabaqabeGadaaakqaaeeqaaiabdMeajnaaBaaaleaacqWGNbWzcqGGSaalcqWGUbGBaeqaaOGaeiikaGIaeqyYdC3aaSbaaSqaaiabdUgaRbqabaGccqGGPaqkcqGH9aqpdaWcaaqaaiabikdaYaqaaiabd6eaobaadaabdaqaamaaqahabaGaemywaK1aaSbaaSqaaiabdEgaNbqabaGccqGGOaakcqWG0baDcqGGPaqkcqWGLbqzdaahaaWcbeqaaiabgkHiTiabdMgaPjabeM8a3naaBaaameaacqWGRbWAaeqaaSGaemiDaqhaaaqaaiabdsha0jabg2da9iabigdaXaqaaiabd6eaobqdcqGHris5aaGccaGLhWUaayjcSdWaaWbaaSqabeaacqaIYaGmaaaakeaacqGH9aqpdaWcaaqaaiabd6eaobqaaiabikdaYaaacqGGOaakcqWGHbqydaqhaaWcbaGaem4zaCMaeiilaWIaem4AaSgabaGaeGOmaidaaOGaey4kaSIaemOyai2aa0baaSqaaiabdEgaNjabcYcaSiabdUgaRbqaaiabikdaYaaakiabcMcaPiabcYcaSiaaxMaacaWLjaWaaeWaaeaacqaI0aanaiaawIcacaGLPaaaaaaa@6A00@

for *k *= 1, ..., *m *and *g *= 1, ..., G
 MathType@MTEF@5@5@+=feaafiart1ev1aaatCvAUfKttLearuWrP9MDH5MBPbIqV92AaeXatLxBI9gBamXvP5wqSXMqHnxAJn0BKvguHDwzZbqegm0B1jxALjhiov2DaebbnrfifHhDYfgasaacH8akY=wiFfYdH8Gipec8Eeeu0xXdbba9frFj0=OqFfea0dXdd9vqai=hGuQ8kuc9pgc9s8qqaq=dirpe0xb9q8qiLsFr0=vr0=vr0dc8meaabaqaciaacaGaaeqabaWaaeGaeaaakeaaimaacaWFhbaaaa@3962@. Under the assumption that *ε*_*gt*_'s are identically independently distributed normal random errors with mean 0 and homogenous variance *σ*^2 ^(that is, *Y*_*g*_(*t*) is a normal white noise), Fisher [7] proposed a G-statistic and derived the exact null distribution of *G*. Suppose it is of our interest to test

*H*_0_: *Y_g_*(*t*) = *μ *+ *ε_gt_*,     (5)

versus

*H*_1_: *Y_g_*(*t*) = *μ *+ *A *cos(*ωt*) + *B *sin (*ωt*) + *ε_gt_*,     (6)

then for a fixed gene *g*, the Fisher's *G*-statistic is given by



For details on the *G *test statistic, its null distribution and the percentage points of the *G *test statistics, please refer to Fisher [7], Davis [18], Wilks [19], and Priestley [20].

Other test statistics for searching "hidden periodicity" in a time series have been proposed as part of spectral analysis (Fuller [21]) in the literature. For the following more general setting of hypothesis testing of

*H*_0_: *Y*_*g*_(*t*) is a normal white noise,     (8)

versus

*H*_0_: *Y*_*g*_(*t*) is not a normal white noise,     (9)

for fixed gene *g*, Bartlett [8] proposed to use a *C*-statistic as a test statistic to fulfill the task of such hypothesis testing procedure. For a fixed gene *g*, we obtain the *C*-statistic as

Cg=max⁡1≤k≤m−1|Cg,k−k/m|     (10)
 MathType@MTEF@5@5@+=feaafiart1ev1aaatCvAUfKttLearuWrP9MDH5MBPbIqV92AaeXatLxBI9gBaebbnrfifHhDYfgasaacH8akY=wiFfYdH8Gipec8Eeeu0xXdbba9frFj0=OqFfea0dXdd9vqai=hGuQ8kuc9pgc9s8qqaq=dirpe0xb9q8qiLsFr0=vr0=vr0dc8meaabaqaciGacaGaaeqabaqabeGadaaakeaacqWGdbWqdaWgaaWcbaGaem4zaCgabeaakiabg2da9maaxababaGagiyBa0MaeiyyaeMaeiiEaGhaleaacqaIXaqmcqGHKjYOcqWGRbWAcqGHKjYOcqWGTbqBcqGHsislcqaIXaqmaeqaaOWaaqWaaeaacqWGdbWqdaWgaaWcbaGaem4zaCMaeiilaWIaem4AaSgabeaakiabgkHiTiabdUgaRjabc+caViabd2gaTbGaay5bSlaawIa7aiaaxMaacaWLjaWaaeWaaeaacqaIXaqmcqaIWaamaiaawIcacaGLPaaaaaa@4EEF@

with



for *g *= 1, ..., G
 MathType@MTEF@5@5@+=feaafiart1ev1aaatCvAUfKttLearuWrP9MDH5MBPbIqV92AaeXatLxBI9gBamXvP5wqSXMqHnxAJn0BKvguHDwzZbqegm0B1jxALjhiov2DaebbnrfifHhDYfgasaacH8akY=wiFfYdH8Gipec8Eeeu0xXdbba9frFj0=OqFfea0dXdd9vqai=hGuQ8kuc9pgc9s8qqaq=dirpe0xb9q8qiLsFr0=vr0=vr0dc8meaabaqaciaacaGaaeqabaWaaeGaeaaakeaaimaacaWFhbaaaa@3962@. Durbin ([9,22]) provided the details of the null distribution of the test statistic *C *under the normality assumption.

According to Fisher [7], the observed significance value, or p-value pgG
 MathType@MTEF@5@5@+=feaafiart1ev1aaatCvAUfKttLearuWrP9MDH5MBPbIqV92AaeXatLxBI9gBaebbnrfifHhDYfgasaacH8akY=wiFfYdH8Gipec8Eeeu0xXdbba9frFj0=OqFfea0dXdd9vqai=hGuQ8kuc9pgc9s8qqaq=dirpe0xb9q8qiLsFr0=vr0=vr0dc8meaabaqaciGacaGaaeqabaqabeGadaaakeaacqWGWbaCdaqhaaWcbaGaem4zaCgabaGaem4raCeaaaaa@30B2@, for the hypothesis testing of the periodicity of a fixed gene *g *using *G*-statistic as the test statistic is expressed as in (1), or again



where *ξ*_*g *_is the sample realization of the *G*-statistic value calculated from (7) divided by m, and *L*(*ξ*_*g*_) is the largest integer less than 1/*ξ*_*g*_. Meanwhile, according to Durbin [9], the p-value, pgC
 MathType@MTEF@5@5@+=feaafiart1ev1aaatCvAUfKttLearuWrP9MDH5MBPbIqV92AaeXatLxBI9gBaebbnrfifHhDYfgasaacH8akY=wiFfYdH8Gipec8Eeeu0xXdbba9frFj0=OqFfea0dXdd9vqai=hGuQ8kuc9pgc9s8qqaq=dirpe0xb9q8qiLsFr0=vr0=vr0dc8meaabaqaciGacaGaaeqabaqabeGadaaakeaacqWGWbaCdaqhaaWcbaGaem4zaCgabaGaem4qameaaaaa@30AA@, for the hypothesis testing of the periodicity of a fixed gene *g *using *C*-statistic as the test statistic is given in (2), or specifically,



where *a*_*g *_= *mC*_*g*_, *C*_*g *_is given in (10), [*a*_*g*_] = *INT*{*a*_*g*_}, and *n *= *m *- 1.

The C&G Procedure utilizes both of the test statistics and gives a practical way for identifying significant periodic genes in massive microarray data.
